# Metabolic network analysis and experimental study of lipid production in *Rhodosporidium toruloides* grown on single and mixed substrates

**DOI:** 10.1186/s12934-015-0217-5

**Published:** 2015-03-18

**Authors:** Rajesh Reddy Bommareddy, Wael Sabra, Garima Maheshwari, An-Ping Zeng

**Affiliations:** Institute of Bioprocess and Biosystems Engineering, Hamburg University of Technology, Denickestrasse 15, D-21073 Hamburg, Germany

**Keywords:** Metabolic network analysis, Elementary mode analysis, Lipids, Glycerol, Biomass, Oleaginous yeast

## Abstract

**Background:**

Microbial lipids (triacylglycerols, TAG) have received large attention for a sustainable production of oleochemicals and biofuels. *Rhodosporidium toruloides* can accumulate lipids up to 70% of its cell mass under certain conditions. However, our understanding of lipid production in this yeast is still much limited, especially for growth with mixed substrates at the level of metabolic network. In this work, the potentials of several important carbon sources for TAG production in *R.toruloides* are first comparatively studied *in silico* by means of elementary mode analysis followed by experimental validation.

**Results:**

A simplified metabolic network of *R.toruloides* was reconstructed based on a combination of genome and proteome annotations. Optimal metabolic space was studied using elementary mode analysis for growth on glycerol, glucose, xylose and arabinose or in mixtures. The *in silico* model predictions of growth and lipid production are in agreement with experimental results. Both the *in silico* and experimental studies revealed that glycerol is an attractive substrate for lipid synthesis in *R. toruloides* either alone or in blend with sugars. A lipid yield as high as 0.53 (C-mol TAG/C-mol) has been experimentally obtained for growth on glycerol, compared to a theoretical maximum of 0.63 (C-mol TAG/C-mol). The lipid yield on glucose is much lower (0.29 (experimental) vs. 0.58 (predicted) C-mol TAG/C-mol). The blend of glucose with glycerol decreased the lipid yield on substrate but can significantly increase the overall volumetric productivity. Experimental studies revealed catabolite repression of glycerol by the presence of glucose for the first time. Significant influence of oxygen concentration on the yield and composition of lipids were observed which have not been quantitatively studied before.

**Conclusions:**

This study provides for the first time a simplified metabolic model of *R.toruloides* and its detailed *in silico* analysis for growth on different carbon sources for their potential of TAG synthesis. Experimental studies revealed the phenomenon of catabolite repression of glycerol by glucose and the importance of oxygen supply on the yield and composition of lipids. More systematic studies are needed to understand the mechanisms which should help to further optimize the lipid production in this strain of industrial interest.

**Electronic supplementary material:**

The online version of this article (doi:10.1186/s12934-015-0217-5) contains supplementary material, which is available to authorized users.

## Background

During the past years fuel production from biomass has gained enormous interest due to the escalating cost and scarcity of fossil fuels. Biodiesel industry has become important and the use of plant oils as raw materials is not ecologically sustainable because of the relatively high production costs and their competition with global supply of food. Despite the expected high production capacity of biodiesel the anticipated target has increased at a slower rate [1], mainly due to its relatively high production cost. However, utilization of glycerol, a co-product from biodiesel production, is one of the promising options for lowering its production cost [2,3]. In fact, biodiesel production generates about 10% (w/w) glycerol as the main byproduct. Therefore, many researchers have worked on valorization of glycerol and successfully produced value added chemicals which are industrially significant [4-8].

On the other hand, microbial lipids are being explored as raw materials for the production of biodiesel and functional oils with comparable properties of those produced from plant oils [9-11]. Moreover, microbial substitutes of industrially important products like cocoa butter from oils produced by microorganism has been previously reported [12]. An important advantage offered by the application of the oleaginous microorganisms is their ability to produce lipids from cheep substrates. Lignocellulosic biomass is considered as the only foreseeable, feasible and sustainable resource for renewable fuel, and large efforts have been implemented worldwide to replace the first generation of fuels based on high-value sugars and oils with 2^nd^ generation biofuels based on cheaper and more abundant lignocellulosic biomass. In fact, several oleaginous microorganisms were reported for efficient lipid production using industrial wastes and biomass hydrolysate [2,13-18]. Since glycerol is the backbone of microbial lipids, it is of interest to examine if glycerol, especially glycerol from biodiesel production, can be effectively used together with biomass hydrolysate. Few studies have been reported on the use of mixed substrates for the synthesis of microbial lipids.

Several yeast strains are known for lipids production, which include *Cryptococcus albidus*, *Lipomyces lipofera*, *Lipomyces starkeyi*, *Rhodosporidium toruloides*, *Rhodotorula glutinis*, *Trichosporon pullulan*, and *Yarrowia lipolytica* [19]. Ageitos et al. [19] compared the different yeast strains in terms of productivity and yield, of which *R. toruloides* and *R. glutinis* were shown to be the most promising. *Y. lipolytica* was also proven to be a potential lipid producer, especially after improvement with genetic tools.

The red yeast *Rhodosporidium toruloides* has a high capability for growth and lipid synthesis on a range of carbon sources from glucose, fructose and xylose to glycerol [20-22]. Lipids can accumulate to a concentration of 60% (*w/w*) in cell mass of *R. toruloides*. Its ability to simultaneously assimilate sugars, especially glucose and glycerol has not been studied in detail. Studies on a closely related species *R. glutinis* showed that glucose and glycerol can be utilized simultaneously with preference toward glycerol which is similar to *Y. lipolytica* [23].

With substrate cost and availability being continually changing, the utilization of multiple feed stocks is crucial for process viability. Therefore the aim of this study was to analyze the metabolic network and fluxes of *R. toruloides* grown on different substrates and substrate mixtures, using detailed biochemical knowledge on genome scale. Elementary mode analysis was used to elucidate the optimal pathway and the various fluxes for lipid accumulation from different substrates. Validation experiments were then performed and the kinetics of growth, lipid production and substrate uptake were evaluated. Experiments with biomass hydrolysate as pure substrates or with glycerol were also compared. Since lipid production is a process requiring intensive reducing power and energy, the control of oxygen supply on lipid accumulation and composition is important and studied in a well-controlled bioreactor system. An understanding of these metabolic processes can open doors for metabolic engineering of this yeast and for process optimization.

## Results and discussion

### *In silico* analysis of triacylglycerol production on different substrates

The large quantity of information featured in public databases, like details about genomes, pathways and proteins were first used for the reconstruction of the metabolic network and the *in silico* modeling of *R. toruloides*. With the genome annotation and other metabolic capacities in available literature and experiments, a draft metabolic network model was validated and curated manually until a high-quality metabolic network model of *R. toruloides* was constructed (see Methods section and Additional file 1: Table S1 for details). On that basis, the influence of different substrates on lipid production was first investigated. Elementary flux modes were estimated for the defined metabolic network of *R. toruloides* grown on four different substrates (glucose, glycerol, xylose and arabinose). The numbers of elementary modes obtained from the metabolic network for each substrate are shown in Table 1. Maximum theoretical yields of TAG were found to be in the modes where there is no cell mass production. This can be meaningful as lipid production is induced by nitrogen limitation where growth limitation occurs. Among the substrates, the glycerol utilizing network yielded more EMs and a higher theoretical maximum yield of TAG whereas among the sugars glucose showed the highest yield. Comparison of the solution spaces spanned by elementary modes on these substrates is represented in Additional file 1: Figure S1.

**Table 1:** **Elementary modes (EMs) of the metabolic network of Rhodosporidium toruloides for growth on different substrates with corresponding maximum TAG and cell mass yields**

**Carbon source**	**EMs**	**Maximum yield of TAG [g.g** ^**−1**^ **substrate]**	**Maximum yield of TAG [C-mol.C-mol** ^**−1**^ **substrate]**	**Maximum cell mass yield [C-mol.C-mol** ^**−1**^ **substrate]**
Glucose	14,164	0.30	0.58	0.67
Glycerol	33,970	0.32	0.63	0.73
Xylose	9,476	0.29	0.55	0.66
Arabinose	15,231	0.27	0.53	0.63

**Figure 1:**
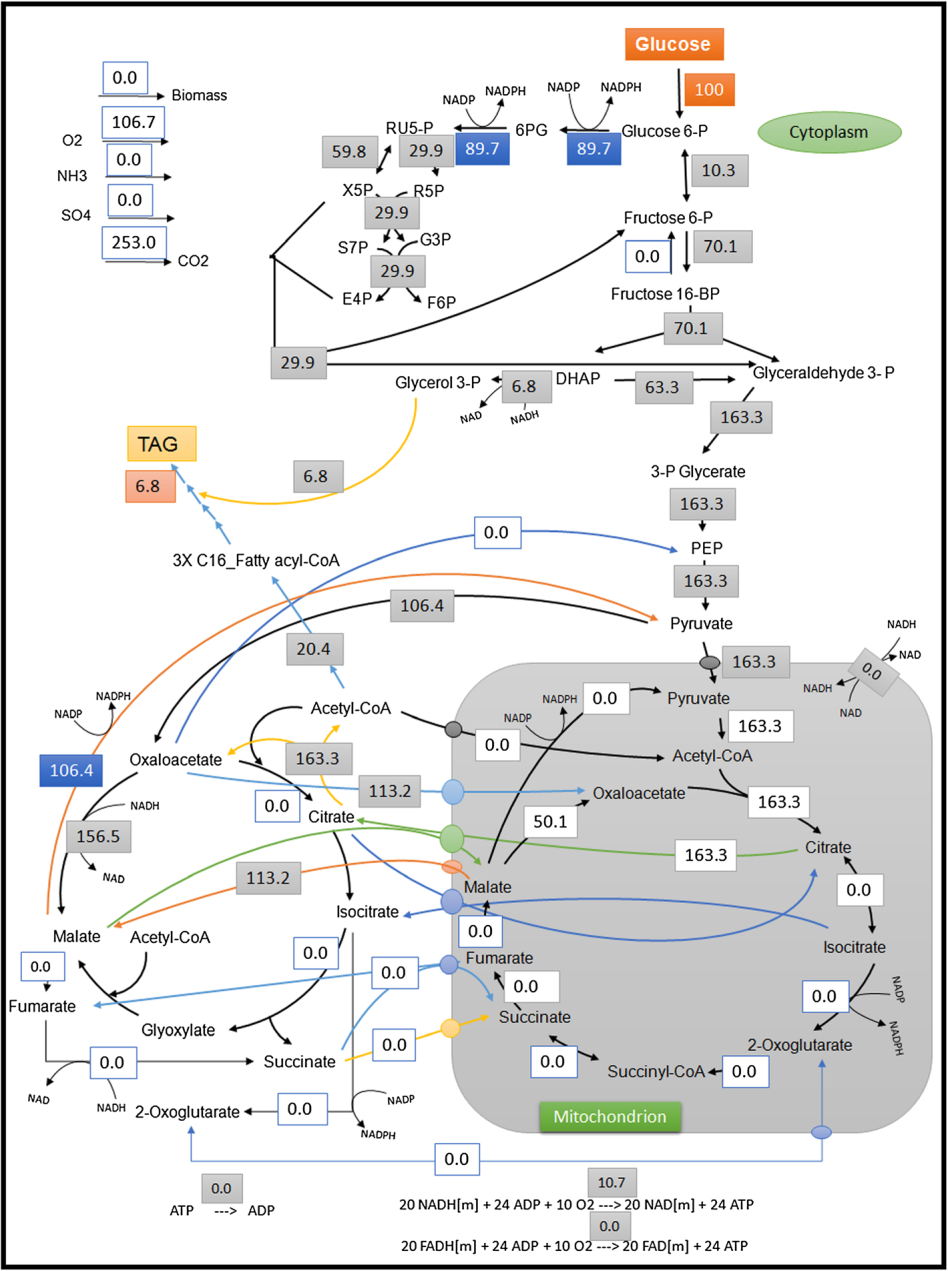
**Optimal flux distribution on glucose.** All values are relative molar fluxes (mmol.g^−1^.h^−1^) normalized to the glucose uptake rate.

With glucose as the sole carbon and energy source, the computed optimum flux distribution is shown in Figure 1. The most common product found in 94% of the modes is cell mass whereas TAG production is found in 25% of the modes. Maximum theoretical yield of the TAG is found to be 0.07 mol.mol^−1^ on the basis of an assumed TAG molecule size of 51 carbons (3X C_16_ palmitic acid + glycerol 3-phosphate). Taking the molecular weight of the assumed TAG (C_51_H_92_O_6_ = 801.27 g.mol^−1^) into account the resulting maximum yield is 0.30 g.g^−1^ glucose. For the production of one mol of TAG, 42 moles of NADPH and 21 moles of ATP are required. From the flux distribution map (Figure 1), 63% of the required NADPH is mainly supplied by the pentose phosphate pathway (PP) and partially by the cytosolic malic enzyme (values normalized to glucose uptake rate as shown in Figure 1). This high flux through the PP pathway might lead to carbon loss in form of CO_2_, but also for a better precursor availability and growth. In fact, 37.2 mol CO_2_ would be released for the synthesis of one mol TAG. Neither the malic enzyme in the cytosol nor the PP pathway alone can compensate for the required NADPH. The flux through the malic enzyme depends on the cytosolic NADH availability as the malic enzyme functions together with the pyruvate-oxaloacetate-malate cycle (POM cycle). About 10% ATP is generated by the respiratory chain reaction using the mitochondrial NADH.

**Figure 2:**
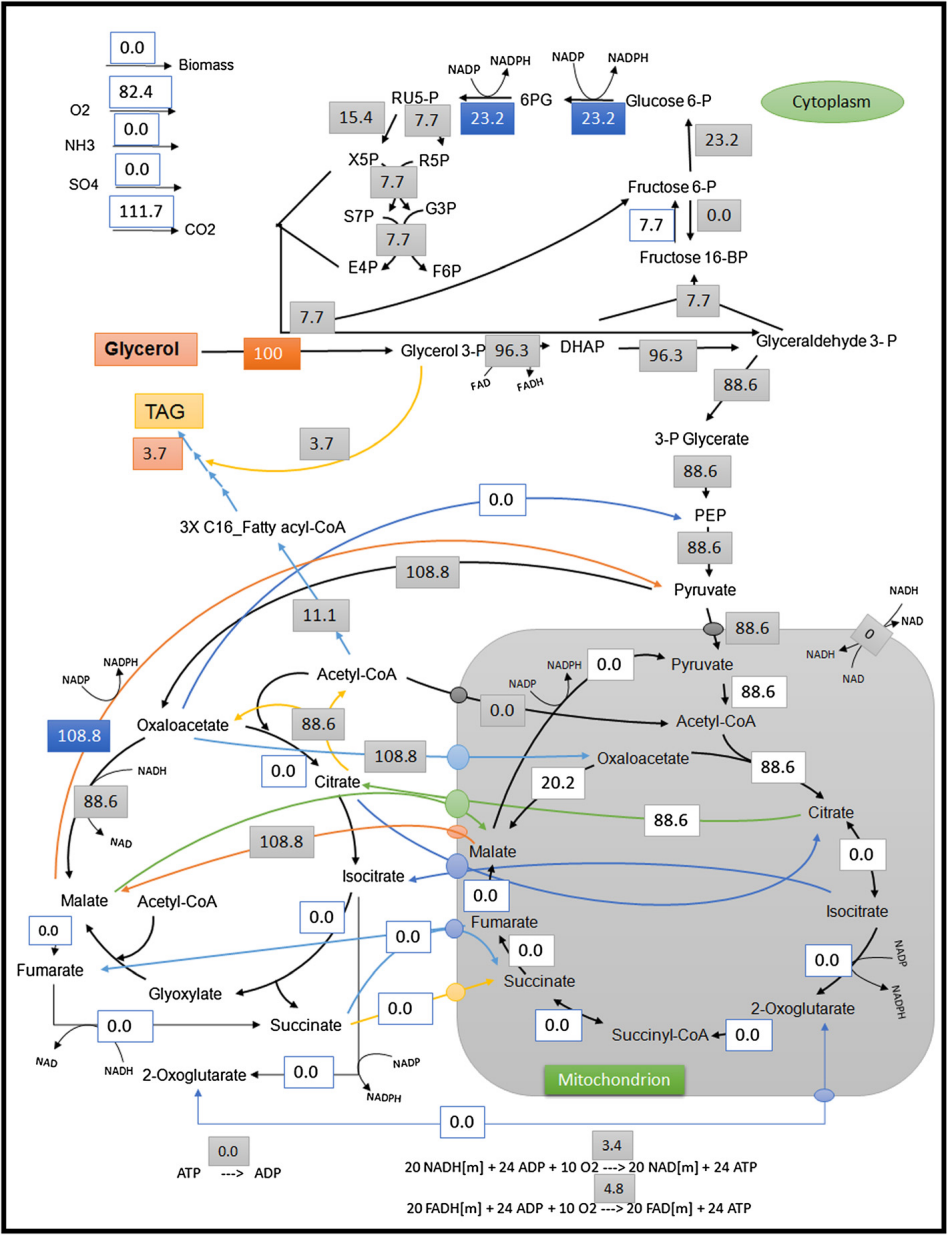
**Optimal flux distribution on glycerol.** All values are relative molar fluxes (mmol.g^−1^.h^−1^) normalized to the glycerol uptake rate.

On the other hand, with glycerol as the sole substrate (Figure 2), the NADPH demand is mainly met by the cytosolic malic enzyme whereas there is a reduced PP pathway flux and hence reduced CO_2_ formation. Glycerol is channeled into glycolysis via the dihydroxyacetone to glyceraldehydes 3-phosphate. Most of the fluxes pass through the lower part of the glycolysis into the pyruvate dehydrogenase complex. Two more optimal modes (data not shown) were identified where similar maximum TAG yields were observed with a succinate to fumarate interconversion in the mitochondrion and cytoplasm. The required ATP is also generated by both the respiratory chain reactions in the mitochondrion.

Similarly, the optimal flux distributions with xylose and arabinose as substrates are shown in Additional file 1: Figures S2 and S3, respectively in the Supplement. Higher numbers of EMs were observed on arabinose compared to xylose. About 96% of the modes produced cell mass and 23% of the modes showed TAG production on both the pentoses. A very high PP pathway fluxes is observed on both pentoses, obviously due to the requirement of NADPH for uptake of these substrates [24]. Xylose showed slightly higher theoretical yields of TAG than arabinose. Most of the fluxes are channeled through the gluconeogenic glucose 6-phosphate isomerase reaction into the PP pathway on both the pentoses. Similar channeling of fluxes is observed towards the pyruvate dehydrogenase complex on both the substrates. Due to the high demand for NADPH on arabinose, the oxaloacetate transporter flux into the mitochondrion should be inactive whereas on all the other substrates it should be active for maximal TAG synthesis. This accumulation of oxaloacetate may drive a high flux through the malate dehydrogenase (MDH) in the cytosol towards malate which in turn also increases the flux through the cytolsolic malic enzyme on arabinose than on xylose.

**Figure 3:**
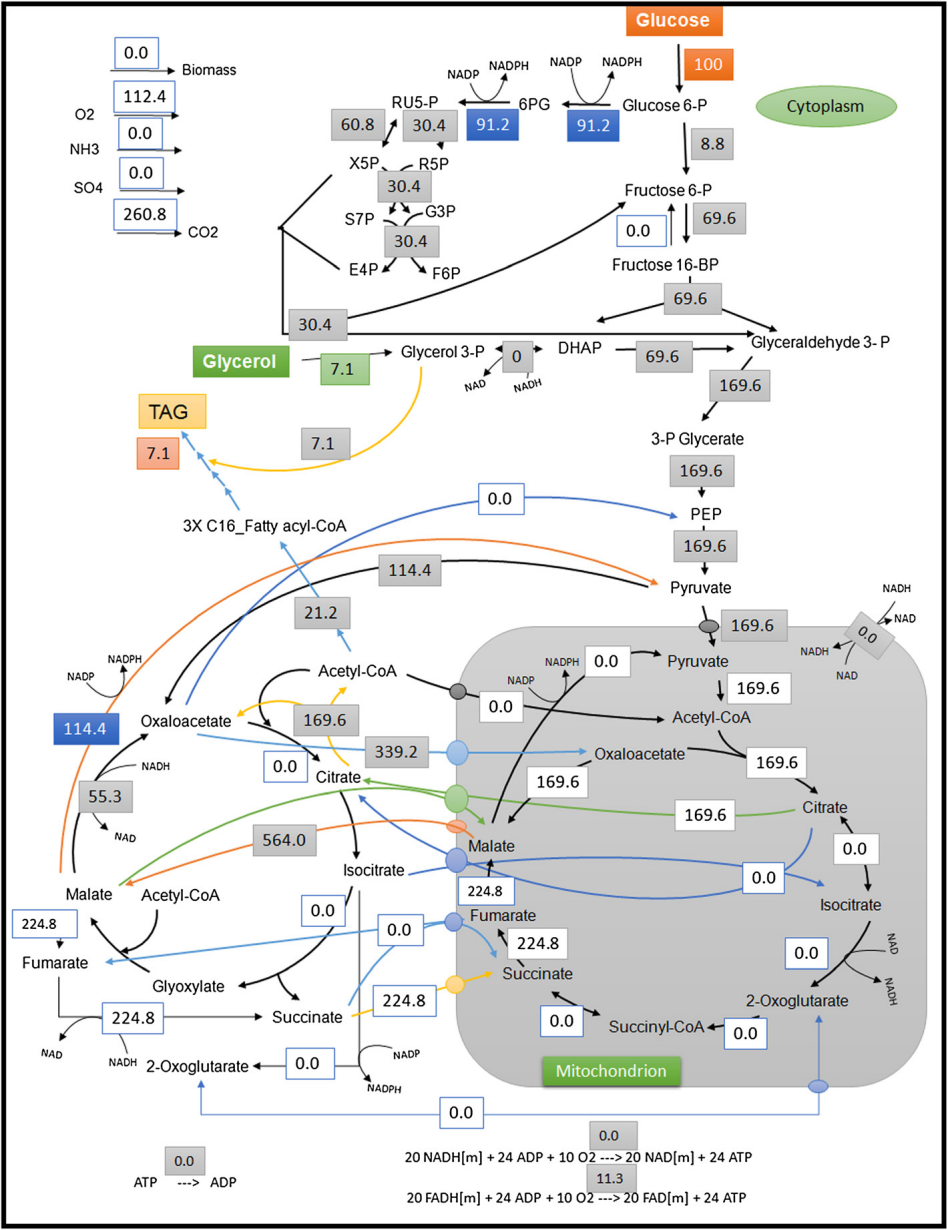
**Optimal flux distribution on glucose and glycerol.** All values are relative molar fluxes (mmol.g^−1^ h^−1^) normalized to the glucose uptake rate.

Using the same method, optimal flux distributions and EFM analysis with the proposed metabolic network of *R. toruloides* were performed using blend of substrates. The maximum TAG yield obtained is 0.58 C_mol_. C_mol_^−1^ substrate with a blend of glucose and glycerol (Figure 3), compared to 0.55 C_mol_. C_mol_^−1^ substrate with a blend of xylose and glycerol (Additional file 1: Figure S4). The represented modes are the best modes consuming both the substrates. Comparing the yields from these analyses, similar maximum TAG yields are observed compared to the yields where single sugars are used (Table 1). Adding glycerol uptake did not show any effect on maximum TAG yields in these *in silico* analyses. CO_2_ produced per mole TAG on different carbon sources are compared from the *in silico* analyses, arabinose as a carbon source has the highest production of CO_2_ per TAG molecule (45.6 mol/mol TAG), whereas glycerol as a sole carbon source has the lowest CO_2_ production (30.7 mol/mol TAG), indicating that glycerol is a desirable substrate in terms of efficiency of carbon utilization. CO_2_ production is proportional to the flux entering into the PP pathway as clearly observed from the *in silico* predictions.

### Controlled bioreactor cultivations of *R. toruloides* and the utilization efficiency of different substrates

**Figure 4:**
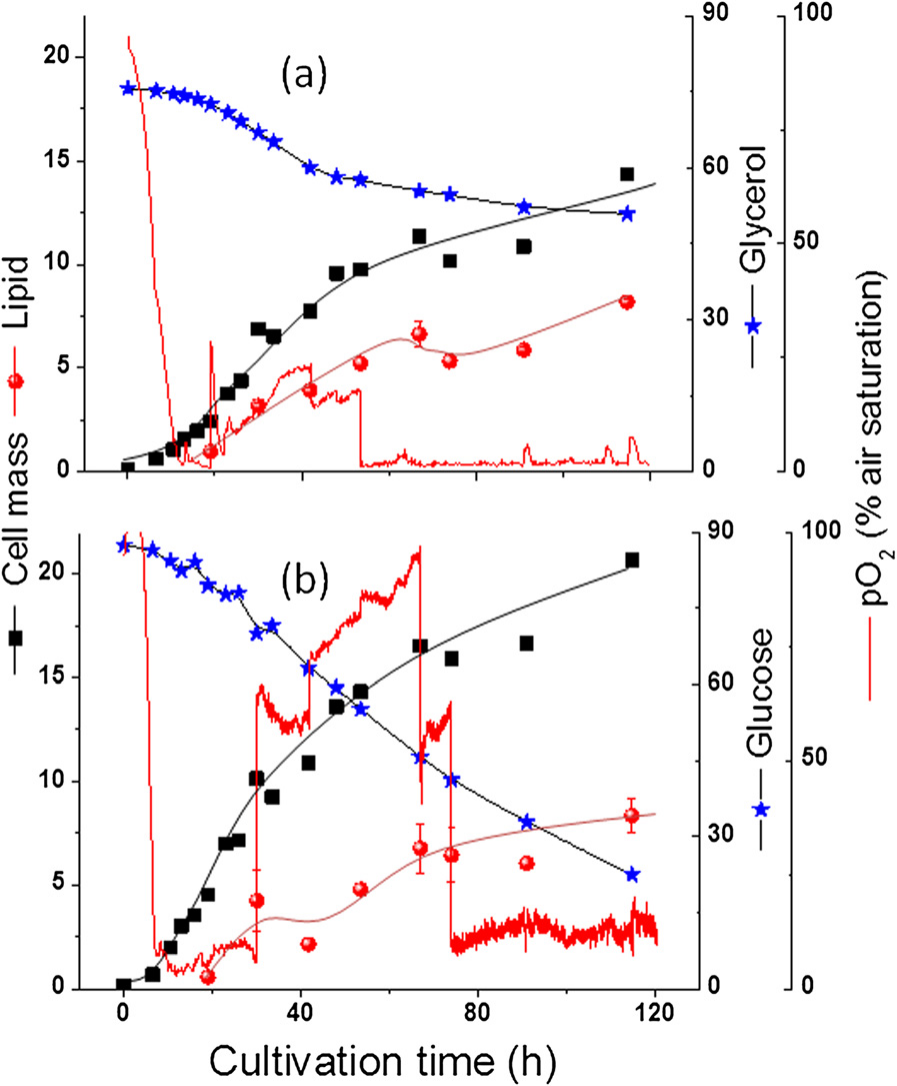
**Batch cultivation of** ***R. toruloides*** **with either glycerol (a) or glucose (b) in pH controlled bioreactor: cell mass, lipid, substrate and pO**_**2**_ **as a function of time.**

To compare the *in silico* predictions regarding the use of different substrates an initial set of cultivations was performed using glucose and glycerol as the sole carbon and energy source. The time profiles of the cultivations are shown in Figure 4. Typically, in the medium used, and with a fixed aeration rate of 0.5 vvm, four distinct culture phases were observed for both substrates: a growth phase under excess of both nutrient and oxygen (10 h or 12 h for growth on glucose and glycerol, respectively), a growth phase under oxygen-limited condition (last for about 10 and 20 h with glycerol and glucose respectively), and a phase with sufficient oxygen supply, presumably due to limitation by other nutrient(s) (which continued for almost 30 h or 40 h for glycerol and glucose respectively), and lastly a phase of oxygen limitation again which continued till the end of the experiment.

The results shown that glucose supported a higher cell mass concentration with a maximum specific growth rate of 0.16 h^−1^ compared to 0.12 h^−1^ with glycerol as the sole carbon source. The lipid concentration on the other hand, was similar in both substrates (~8 g/L, Figure 4). As predicted form the *in silico* analysis, most of the carbon was wasted as CO_2_ and cell mass if glucose was the sole carbon source and lipid accumulation never exceeded 45% (w/w) of cell mass. On the other hand lower cell mass but higher lipid content was observed with glycerol and a maximum lipid content of 57% (on cell mass basis) was achieved. Moreover, the lipid yield on glycerol consumed (0.27 g.g^−1^) is more closer to the theoretical maximum value (0.32 g.g^−1^) predicted than the lipid yield on glucose (0.12 g.g^−1^ experimentally determined vs. 0.30 g.g^−1^ of theoretical maximum). It should be emphasized that the maximum yield of TAG given in Table 1 was calculated for an optimal flux distribution where there is no cell mass production. Nevertheless, it is clear that an optimization of yield is especially needed for growth on glucose. Table 2 shows a comparison between the yields obtained by different *R. toruloides* strains on different substrates and conditions. The lipid yield on glucose could be improved by proper process control, especially by applying nitrogen or phosphorous limitations as demonstrated in literature. We found in this work that the lipid yield on glucose can be significantly improved by using blend with glycerol.

**Table 2:** **Experimental lipid yields and productivity of different** ***R. toruloides*** **strains grown on different substrates**

**Carbon source**	**Lipid productivity**	**Condition**	**Lipid yield** ^*^ **(g/g substrate)**	**Lipid yield (C-mol.C-mol** ^**−1**^ **substrate)**	**Reference**
	**(g/L/h)**				
glucose	0.15	Batch- N limiting, pO2 controlled	0.15	0.29	This work
glycerol	0.06	Batch- N limiting, pO2 controlled	0.24	0.47	
glucose + glycerol	0.12	Batch- N limiting, pO2 controlled	0.17	0.33	
glucose	0.07	Batch- N limiting, pO2 un-controlled	0.12	0.23	
glycerol	0.06	Batch- N limiting, pO2 un-controlled	0.27	0.53	
glucose + glycerol	0.07	Batch- N limiting, pO2 un-controlled	0.2	0.38	
Glucose + glycerol + xylose	0.08	Batch- N limiting, pO2 controlled	0.22	0.42	
(10:20:30)					
Biomass hydrolysate + glycerol	0.09	Batch- N limiting, pO2 controlled	0.17	--	
(glucose- xylose -glycerol =10:20:30)					
glucose	-	Batch, phosphorous limiting	0.21	--	[17]
glucose	-	Continuous- nitrogen limiting	0.19	--	[38]

TAG molecule with 51 carbons and a molecular weight of 801.27 g/mol are used to calculate the yields in C-mol/C-mol.

*lipid yield was calculated from the slope between lipid concentration against consumed sugar(s).

ATP requirements for growth, lipid production and maintenance are supplied mainly through the TCA cycle in the mitochondria. In fact, our *in silico* analysis was all done with a fixed P:O ratio of 1.2. The effects of oxidative phosphorylation (with varied P:O ratio) on TAG yields using glucose as a substrate were analyzed using the proposed model. The change in P:O ratios (from 1.2 to 2) neither showed significant effect on the TAG yields (from 0.58 to 0.60 C_mol_.C_mol_^−1^ glucose), nor cell mass yield (0.67 to 0.71 C_mol_.C_mol_^−1^ glucose), respectively. Hence, it can be concluded that ATP generated by oxidative phosphorylation is not a major limiting factor for TAG synthesis. In agreement with the *in silico* analysis, controlling the oxygen concentration at 50% air saturation in the fermentation broth of *R. toruloides* grown either on glucose or glycerol throughout the cultivation process did not have any significant effect on the yield (Table 2). Similar to the cultivations without pO_2_ control (e.g. O2 limitation in phases 2 and 4, see Figure 4), the maximal growth rate with glucose was 0.23 h^−1^ compared to 0.13 h^−1^ with glycerol in pO_2_ controlled cultivations. The maximum lipid content on glycerol was 61% vs. 57% in pO_2_ uncontrolled culture, whereas it was 48% on glucose vs. 41% in pO_2_ uncontrolled culture. The lipid productivity increased significantly in the pO_2_ controlled experiments, mainly because of the increased growth rates.

**Figure 5:**
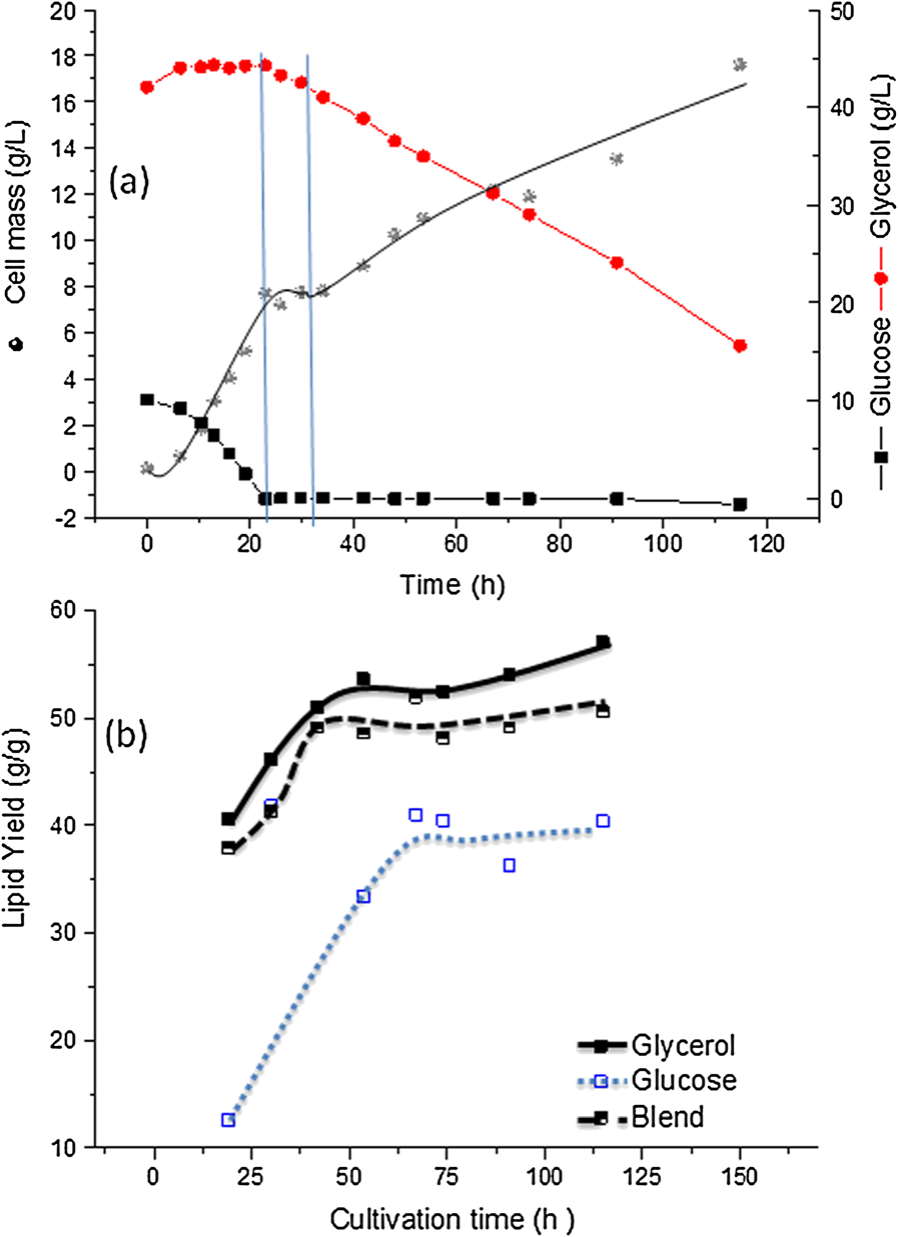
**pO**_**2**_ **uncontrolled cultivation of** ***R. toruloides*** **and the diauxy growth behavior on a mixture of glucose and glycerol (10:90 g/g (a)), and a comparison of the different substrate and blend on the lipid yield on produced cell mass (b).**

Blends of sugars and glycerol were also tested for the production of lipid by *R. toruloides*. Interestingly, this strain showed a catabolite repression in the presence of glucose as depicted in Figure 5a. Catabolite repression was not observed before in certain oleaginous yeasts. Strains of *Y. lipolytica and R. glutinis* were shown previously to metabolize both carbon sources simultaneously [23,25,26].

As shown in Figure 5a, a clear diauxic growth was observed in the mixed substrate cultivations: glycerol was consumed only after glucose consumption in this case. Despite of the observed catabolite repression, the addition of glycerol enhanced the lipid yield on glucose significantly (Figure 5b), confirming the predictions from the *in silico* analysis. In fact, the mixed substrate cultivation has the dual advantages of efficient cell mass production (normally maximized with glucose) with higher lipid content (obtained normally with glycerol). The lipid yields on the substrate consumed and the specific lipid productivities on cell mass are higher when glucose and glycerol are used in mixture rather than only glucose (Table 2).

**Figure 6:**
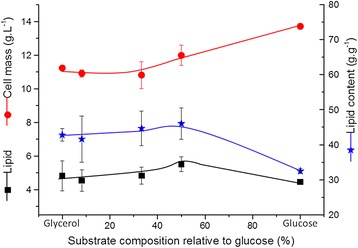
**Cell mass and lipid production by** ***R. toruloides*** **grown on mono-substrate and on different variation of dual substrate fermentation.**

In the above blend substrate fermentation experiments, glucose was used at a concentration of 10% of the added carbon source. The effect of different combinations of the substrates on the lipid yield was further investigated in flask experiments. As compared to mono-substrate fermentation with glucose, a 26% increase of lipid yield was observed if glycerol is added (Figure 6). Similarly to the above results from controlled bioreactors, glucose as mono-substrate supported the highest cell mass production (13.7 g.L^−1^) with the lowest lipid content (32%). Similarly, with a closely related yeast strain *R. glutinis*, Easterling [23] reported that blend substrate fermentation produced more cell mass than when a single carbon source was provided. They also reported that the lipid content was minimum with glucose and significantly higher with either glycerol or blend of both. However, they did not report catabolite repression for *R. glutinis*. However, it should be stressed that, on mixtures of carbohydrates and with the sequential utilization of sugars, the sugar is being consumed with more nitrogen available (and hence more cell mass formation), followed by glycerol consumption during a period of nitrogen restricted growth which will have a positive effect on the lipid yield. Still, the mechanism of the positive role of glycerol for the lipid synthesis of cells grown in blends deserves more detailed studies. Glycerol can be utilized as a source of carbon and energy. But under certain conditions (e.g. in pO2-controlled culture with glucose as the sole carbon source), *R. toruloides* can produce glycerol extracellularly up to 5 g/L (data not shown). The two most important functions of glycerol synthesis in yeast are related to redox balancing and the hyperosmotic stress response [27]. In *Saccharomyces cerevisiae,* production of glycerol occurs to maintain the redox balance. Interestingly, glycerol production is found to be higher in a minimal medium than in a complex medium [28]. It was concluded that in a minimal medium the *de novo* synthesis of amino acids from glucose and ammonia leads to an excess of NADH which is re-oxidized by glycerol formation. In osmotolerant yeasts, glycerol plays a crucial role in their survival [29]. Recently, Petelenz-kurdziel et al. [30] reported that *S. cerevisiae* adapts to hyperosmotic stress by activating the HOG signaling cascade, which controls glycerol accumulation, production and import. Moreover, it was also shown that glycerol transport depends on the strain and the growth conditions. Glycerol production has been never reported in *R. toruloides,* and hence transcriptomic analysis is being further performed in our laboratory.

**Figure 7:**
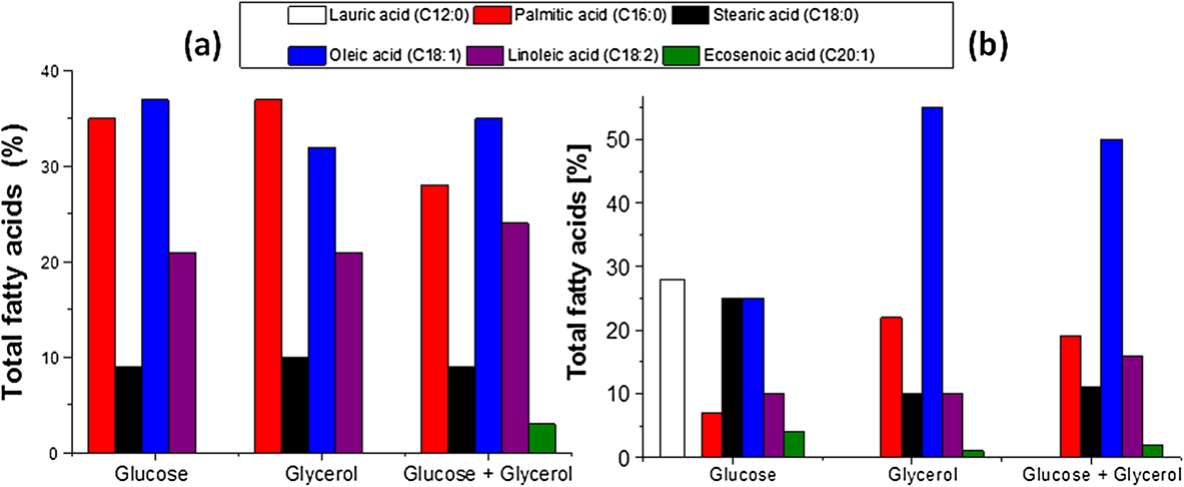
**Fatty acid composition of lipid produced by** ***R.toruloides*** **grown on glucose or glycerol and on mixture. a**. Fatty acid composition from a pO2 controlled cultivation, **b**. fatty acid composition on a pO2 un-controlled cultivation.

The effects of different growth substrates and culture conditions on the fatty acid composition were also studied (Figure 7). In oxygen controlled conditions at pO_2_ of 50% of air saturation, and on all the substrates and blends used, palmitic, oleic and linoleic acid constitute more than 90% of the total fatty acids. In oxygen un-controlled cultivation (constant aeration and agitation, pO_2_ not controlled), about 65% of the fatty acid measured is unsaturated (oleic acid and linoleic acid) if glycerol or blend are used, compared to 57% of saturated fatty acids (lauric acid, stearic acid and palmitic acid) if glucose is the sole carbon source. Oxygen limitation has significantly influenced the fraction of saturated and un-saturated fatty acid composition in the lipid produced by *R.toruloides* especially on glycerol or mixed substrates. Oxygen controlled conditions derived fatty acid compositions that are suitable for biodiesel conversion [9] whereas oxygen un-controlled conditions produced significant amounts of saturated fatty acids which are close to be substitutes of certain important products like cocoa butter.

*R. toruloides* is a known lipogenic yeast and the results presented here confirm its ability to accumulate over 60% of its cell mass as lipid. However, lipid accumulation was strongly affected by the nature of the carbon source provided. The recently sequenced and annotated genome of *R. toruloides*, its proteomic analysis and the available protein annotation have opened doors to understand the underlying mechanism of this organism for lipid production [31-33]. In this work, elementary modes were used to decompose the complex metabolic network into its basic functioning units and to identify the potentials of triacyglyceride production on different substrates in *R. toruloides*. Maximum theoretical TAG yields on hexoses (glucose), pentose (xylose and arabionse), glycerol and on mixture of hexoses & glycerol, pentose & glycerol have been estimated (Table 1) and were in general agreement with previously reported theoretical maximum yields [34,35]. As shown from the computed solution span in Additional file 1: Figure S1, the production is still far from the theoretical optimum and the distance between the actual production state on glucose or glycerol and the theoretical optimum suggests an enormous potential for future optimization. *In silico* optimization, identification of limiting steps and finally suggestions for future genetic modification are discussed below.

One of the key processes for fatty acid biosynthesis is the provision of reducing power (e.g. NADPH) to reduce the acetyl group and channel it into the acyl chain of fatty acid. In *Y. lipolytica*, the PP pathway is assumed to be the sole supplier of NADPH for lipid synthesis, since it does not possess a cytosolic malic enzyme [36] and the deletion of mitochondrial malic enzyme did not show any effect on lipid production [37]. In *R. toruloides*, an alternative NADPH supplying reaction would be the cytosolic NADP-dependent isocitrate dehydrogenase (ICDH) which is inactive in the predicted optimal flux distribution of glucose (Figure 1). A single gene deletion study of our model was performed to remove the cytosolic malic enzyme from the reactions. A reduction in maximum theoretical yield of TAG was observed (from 0.30 g.g^−1^ to 0.27 g.g^−1^) on glucose and glycerol (0.32 g.g^−1^ to 0.27 g.g^−1^). However, the theoretical maximum yield retained to 0.30 g.g^−1^ on glucose and 0.32 g.g^−1^ on glycerol if a NADP-dependent acetaldehyde dehydrogenase (ALD6) is incorporated. This enzyme constitutively catalyzes the conversion of acetaldehyde to acetate, generating an NADPH in the cytoplasm. Another alternate route for supplying NADPH as proposed by Ratledge [34] can be a mitochondrial NAD-dependent ICDH acting in the reverse direction. EFM analysis and optimal flux distribution were done (Additional file 1: Figure S5) and the maximum theoretical yield of TAG on glucose is retained to 0.30 g.g^−1^ glucose. A third possible target could be a heterologous NADPH generating enzyme or an endogenous engineered NADPH generating enzyme, both in the glycolytic pathway [38] which could be a potential source for NADPH. By changing GAPDH, which is a NAD-dependent glyceraldehyde dehydrogenase in the glycolytic pathway, to an NADP-dependent enzyme can increase the theoretical TAG yields by 7% on glucose and 9% on glycerol.

Transhydrogenases for converting NADH to NADPH could be another alternative source for enhancing the availability of NADPH. Nevertheless, oleaginous yeasts or fungi have never been reported to harbor a transhydrogenase. Bacterial cells on the other hand, possess transhydrogenase activity. Recent genome sequencing and annotation has predicted a NADP-transhydrogenase like protein (RTG_03342) in *Rhodotorula glutinis* ATCC 204091 [39] with a UniProtKB id *GOT2B1*. This protein is 84% identical to an uncharacterized protein of *R. toruloides* (RHTO_06438). A theoretical analysis of the metabolic implications of a transhydrogenase reaction was investigated using the *R. toruloides* model. About 10% higher theoretical maximum yields were calculated on all the substrates when a transhydrogenase activity was introduced (Table 3). The optimal modes with the highest lipid yield do not include PP pathway flux but a high flux through the glycolysis is found which generates the cytosolic NADH for conversion to NADPH through the transhydrogenase reaction. On all sugars about 10% of the flux is also observed for the cytosolic NADP-dependent ICDH providing NADPH other than the transhydrogenase for TAG synthesis. On glycerol the sole NADPH supplier is found to be the transhydrogenase reaction. With the aid of already established genetic engineering techniques [40] for *R.toruloides*, the predicted modifications should facilitate the development of superior lipid producing strains.

**Table 3:** **Elementary modes (EMs) and theoretical TAG yields with heterologous genes/reactions**

**Carbon source**	**EMs**	**Characteristics**	**Maximum yield of TAG [C-mol.C-mol** ^**−1**^ **substrate]**	**Maximum yield of TAG [g.g** ^**−1**^ **substrate]**
Glucose	30,132	Transhydrogenase	0.64	0.33
Glycerol	57,172	Transhydrogenase	0.68	0.35
Xylose	21,323	Transhydrogenase	0.64	0.33
Arabinose	27,264	Transhydrogenase	0.64	0.33
Glucose	26,878	Cytosolic NADP-dependent GAPDH	0.62	0.32
Glycerol	42,578	Cytosolic NADP-dependent GAPDH	0.67	0.35
Glucose	6858	Without cytosolic Malic enzyme	0.51	0.27
Glucose	12,487	With NADP-dependent ALD6	0.58	0.30
Glycerol	23,658	With NADP-dependent ALD6	0.63	.032
Glucose	15,331	Cytosolic NADH source	0.68	0.36
Glycerol	35,804	Cytosolic NADH source	0.68	0.35

## Conclusions

The present work describes the successful application of reconstruction of metabolic network and it’s *in silico* analysis for determining and understanding the potential and metabolic pathways for lipid production in *R. toruloides* grown on different substrates, especially for substrate blends. Cultivations of *R. toruloides* were carried out with different substrates and in a well-controlled bioreactor system. The experimental results are in general agreement with model predictions in terms of lipid yield.

The constructed metabolic model can be used to guide further optimization of lipid synthesis in this strain. The maximal lipid yield experimentally achieved is still significantly below the theoretical maximum, especially for growth on glucose (0.29 vs. 0.58 C-Mol/C-Mol) and its blend with glycerol. Several strategies to increase the lipid yield are identified and discussed with the help of the metabolic model.

## Methods

### Microorganism and media

*R. toruloides* DSMZ 4444 was used for the current study. The strain was maintained at −80°C on potato dextrose medium with 20% glycerol (v/v). The medium for seed cultures and main cultures was a nitrogen limited medium which is similar to that reported previously [26]. Carbon sources (glucose, glycerol, xylose, arabinose or biomass hydrolysates) were autoclaved separately and added together with sterile FeCL_3_ and CaCl_2_ solutions and inoculated immediately. Spruce BH (Borregard, Norway) was also used in co-substrate fermentation with glycerol. An enzymatic hydrolysis of spruce was done without buffer. The hydrolysate was then heated at 80°C for 15–20 min to inactivate the enzymes. The supernatant was removed and filtered with a centrifuge with filter bag. The sample was then concentrated by vacuum evaporation at 60°C. The concentrated hydrolysate contained 550 g/L glucose and 35 g/L xylose.

### Cultivations

Seed cultures and cultivations in flasks were performed in baffled shake flasks incubated at 30°C and 180 rpm for 24 h. Batch cultivations were performed in a 1.5 L well-equipped parallel bioreactor system (DASGIP parallel bioreactor system, Jülich, Germany) with 1 L initial working volume. Cultivations were started by inoculating 30 mL (3%) from the seed cultures grown for 24 h. pH was maintained at 6.0 using 5 N NaOH and 2 M HCL. Dissolved oxygen was maintained at 50% air saturation. Carbon dioxide evolution rates and oxygen uptake rates were automatically evaluated by the online DASGIP off gas analyzing system equipped with sensors from Bluesens.

### Analytical methods

Cell growth was recorded as optical density at 600 nm. Cell mass was harvested during the cultivations after centrifugation (5000 rpm, 10 min at 4°C). Cell dry weight was determined gravimetrically after drying the harvested cells in an oven at 80°C to a constant weight. Extraction of lipids was performed with a modified method described previously using Folsch solution (Chloroform: Methanol = 2:1 vol.vol^-1,^ [41]. Non-lipid cell mass was calculated after subtraction of intracellular lipids from the total cell mass. Quantification of glucose, glycerol and organic acids was carried out using high-performance liquid chromatography (HPLC; Kontron Instruments, United Kingdom) with separation on an Aminex HPX-87H column at 60°C with 0.005 M H_2_SO_4_ and detection via refractive index or by UV absorption at 210 nm. Ammonia concentration in the supernatant was determined by photometric measurements using a kit from Macherey Nagel, Germany. GC analysis of the fatty acid methyl esters was performed as reported [42] with a Varian 3900 gas chromatograph equipped with a flame ionization detector (FID) and a TR-FAME column (Thermo Scientific, Germany, 50 m X 0.22 mm X 0.25 μm).

### Metabolic network and elementary mode analysis

**Figure 8:**
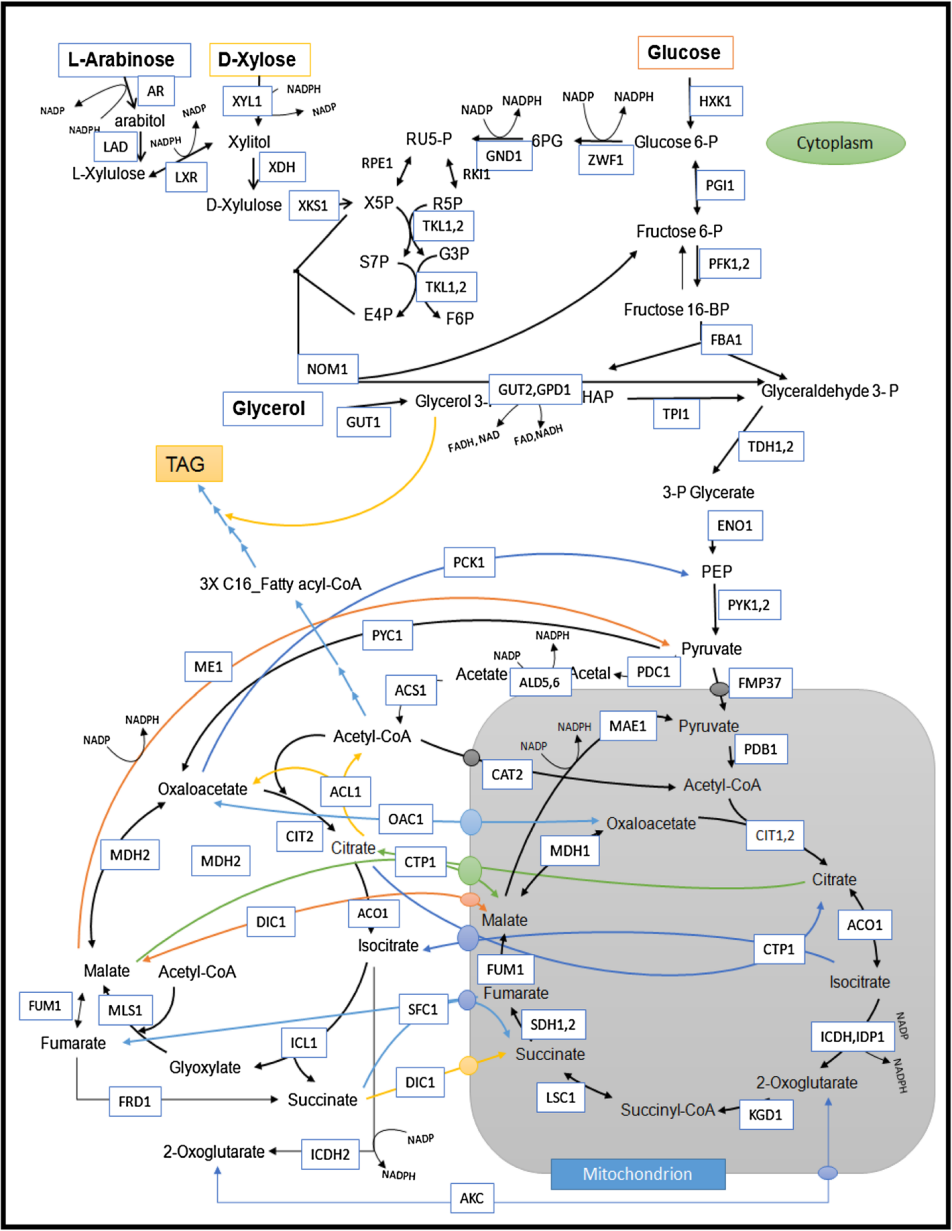
**Metabolic model of Triacylglycerol producing** ***Rhodosporidium toruloides*** **.**

A metabolic network with the most important reactions of *R. toruloides* was reconstructed and is depicted in Figure 8. A complete list of the considered reactions is represented in the Additional file 1: Table S1. The metabolic network model was built manually by considering the available genome sequence and proteomic evidences [33] from UniProtKB. Briefly, the model includes all relevant pathways of central carbon metabolism comprising of glucose, glycerol, xylose and arabinose uptakes. Triacylglycerol (TAG) synthesis is assumed to be formed from glycerol and C_16_ equivalent fatty acids (i.e. palmitic acid) as shown in the reaction network (Additional file 1: Table S1). Assuming that the TAG (C57, M.W = 885.4 g/mol) is formed from glycerol and C_18_ oleic acid, it did not show any difference in the yields from that were observed based on C_16_ palmitic acid assumption. So, C_16_ palmitic acid was used for analysis for all the considered *in silico* predictions. Cellular compartments mitochondrion and cytosol were considered together with respective transport reactions. For elementary mode analysis (EFM), the influx of substrates (glucose, glycerol, xylose, arabinose, oxygen, nitrogen and sulphur) and efflux of products (triacylglyceride, cell mass, carbon dioxide and ATP for maintenance) are considered. The P/O ratio is taken as 1.2 and the cell mass equation is taken from *S.cerevisiae’s* data [43]. EFMs were calculated using the software tool CellNetAnalyzer [44] in MATLAB 2013a. The EFMs were sorted and the theoretical yields on different substrates were calculated using Microsoft Excel. EFMs correspond to minimal functional pathways of a metabolic network and are useful to study various functional network properties [45,46].

The metabolic network with D-glucose as a substrate comprises of 69 reactions, of which 27 are reversible with 61 internal metabolites and 8 external metabolites. With glycerol as a sole carbon source, the network is comprised of 71 reactions (28 reversible) of which 61 are internal and 8 external metabolites. The network with D-xylose and L-arabinose comprises 71 and 73 reactions respectively. Theoretical maximum yields were calculated based on the obtained fluxes. All fluxes are given in mmol.g^−1^.h^−1^ normalized to the substrate uptake rate.

## Additional file

Additional fie 1: Table S1.
*In silico* pathway analysis and lipid production of *Rhodosporidium toruloides* on mixed substrates. **Figure S1.** The biomass yield [c-mol.c-mol^-1^] on corresponding substrates is plotted against the TAG yield on the substrate which is in c-mol.c-mol^-1^. Theoretical maximum yields on each substrate with and without biomass formation are represented. **Figure S2.** Optimal flux distribution on xylose. All values are relative molar fluxes (mmol.g^-1^h^-1^) normalized to the xylose uptake rate. **Figure S3.** Optimal flux distribution on arabinose. All values are relative molar fluxes (mmol.g^-1^h^-1^) normalized to the arabinose uptake rate. **Figure S4.** Optimal flux distribution on xylose and glycerol. All values are relative molar fluxes (mmol.g^-1^h^-1^) normalized to the xylose uptake rate. **Figure S5.** Optimal flux distribution on glucose with ICDH acting in reverse direction and without cytosolic malic enzyme. All values are relative molar fluxes (mmol.g^-1^h^-1^) normalized to the xylose uptake rate.
