# Supplementary material for: Metabolic network analysis and experimental study of lipid production in Rhodosporidium toruloides grown on single and mixed substrates
Source: Microb Cell Fact. 2015 Mar 18;14:36. doi: 10.1186/s12934-015-0217-5 (PMC4377193; doi:10.1186/s12934-015-0217-5)
Supplement: Additional fie 1: Table S1. — In silico pathway analysis and lipid production of Rhodosporidium toruloides on mixed substrates. Figure S1. The biomass yield [c-mol.c-mol-1] on corresponding substrates is plotted against the TAG yield on the substrate which is in c-mol.c-mol-1. Theoretical maximum yields on each substrate with and without biomass formation are represented. Figure S2. Optimal flux distribution on xylose. All values are relative molar fluxes (mmol.g-1h-1) normalized to the xylose uptake rate. Figure S3. Optimal flux distribution on arabinose. All values are relative molar fluxes (mmol.g-1h-1) normalized to the arabinose uptake rate. Figure S4. Optimal flux distribution on xylose and glycerol. All values are relative molar fluxes (mmol.g-1h-1) normalized to the xylose uptake rate. Figure S5. Optimal flux distribution on glucose with ICDH acting in reverse direction and without cytosolic malic enzyme. All values are relative molar fluxes (mmol.g-1h-1) normalized to the xylose uptake rate. [file 12934_2015_217_MOESM1_ESM.docx]

| **Enzyme description**  **Supplemetary file: *In silico* pathway analysis and lipid production of *Rhodosporidium toruloides* on mixed substrates**  Metabolic network of *R.toruloides:* The metabolic network of *R.toruloides* has been constructed considering the current knowledge from literature [Liu et al.,(2009), Zhu et al.,(2012) and Kumar et al.,(2012)] and from protein database UniProtKB. Biomass equation is taken from (Gruchattka et al., (2013)). | **ORF/UniProtKB ID** | **Reaction** |
| --- | --- | --- |
| **Influx** | | |
| Hexose transporter (HXT) | RHTO_03448;RHTO_07706 | --> Gucose[e] |
| Transport |  | --> Glycerol[e] |
| Transport |  | --> Xylose[e] |
| Transport |  | --> Arabinose[e] |
| Transport |  | --> NH3[c] |
| Transport |  | --> SO4[c] |
| Transport |  | --> O2 |
| **Efflux** | | |
| TAG export |  | TAG[c] --> |
| Growth (mmol/gDCW*h) |  | - 1. biomass[c] --> |
| ATP for maintenance |  | ATPmaintenance[c] --> |
| Carbondioxide excretion |  | CO2 --> |
| **Glycolysis** | | |
| Hexokinase (HXK1) | RHTO_06870/M7X0J8 | GLC[c] + ATP --> G6P[c] + ADP |
| Glucose-6-phosphate isomerase (PGI1) | RHTO_04058/M7WNZ9 | G6P[c] <==> F6P[c] |
| 6-phosphofructokinase  (PFK1) | RHTO_00494/M7X6S3 | F6P[c] + ATP --> FBP[c] + ADP |
| Fructose-bisphosphate aldolase (FBA1) | RHTO_03043/M7X5F4 | FBP[c] <==> GAP[c] + DHAP[c] |
| Triosephosphate isomerase (TPI1) | RHTO_01329/M7WME7 | DHAP[c] <==> GAP[c] |
| Glyceraldehyde 3-phosphate dehydrogenase (TDH) | RHTO_03746/M7WI96 | GAP[c] + NAD[c] <==> 13-PG[c] + NADH[c] |
| Glycerol-3-phosphate dehydrogenase (GPD1) | RHTO_02273 /M7WSY9 | DHAP[c] + NADH[c] <==> NAD[c] + GLYC3P[c] |
| Phosphoglycerate kinase (PGK1) | RHTO_00033/M7X689 | ADP + 13-PG[c] <==> ATP + 3-PG[c] |
| Phosphoglycerate mutase (GPM1) | RHTO_03049/M7X4R8;  RHTO_04793/M7XL58 | 3-PG[c] <==> 2-PG[c] |
| Enolase(ENO1) | RHTO_00323/M7X749 | 2-PG[c] <==> PEP[c] |
| Pyruvate kinase (PYK1) | RHTO_01610/ M7WUI5 | PEP[c] + ADP --> PYR[c] + ATP |
| **Pentose phosphate pathway** | | |
| Glucose -6-phosphate 1- dehydrogenase (ZWF1) | RHTO_07853/ M7WR01 | G6P[c] + NADP[c] --> 6-P-Gluconate[c] + NADPH[c] |
| 6-phosphogluconate dehydrogenase (GND) | RHTO_02788/ M7X3Z4 | 6-P-Gluconate[c] + NADP[c] --> RIB-5P[c] + CO2 + NADPH[c] |
| Ribose 5-phosphate isomerase A (RKI1) | RHTO_06311/ M7WUP8 | Ribulose-5-P[c] <==> Ribose-5-P[c] |
| Ribulose-phosphate 3-epimerase (RPE1) | RHTO_05984/ M7XEA2 | Ribulose-5-P[c] <==> Xylulose-5-P[c] |
| Transketolase (TKL1,2) | RHTO_03248/M7XNL9 | Ribulose-5-P [c] + Xylulose-5-P[c] <==> GAP[c] + S7P[c] |
| Transaldolase (NOM1) | RHTO_06955/M7X0R7 | GAP[c] + S7P[c] <==> E4P[c] + F6P[c] |
| Transketolase (TKL1,2) | RHTO_03248/M7XNL9 | E4P[c] + Xylulose-5P[c] <==> GAP[c] + F6P[c] |
| **TCA cycle** | | |
| Pyruvate dehydrogenase complex (PDB, LAT1) | RHTO_03543/M7WIG9 | PYR[m] + NAD[m] --> AcCoA[m] + NADH[m] + CO2 |
| Citrate synthase (CIT1) | RHTO_07345/M7WZV2 | AcCoA[m] + OAA[m] --> CIT[m] |
| Aconitate hydratase 1(ACO1) | RHTO_00539/M7X6X3;  RHTO_08030/M7WQ73 | CIT[m] <==> ICI[m] |
| NAD-Isocitrate dehydrogenase (IDH1) | RHTO_01289/M7XE28 | ICI[m] + NAD[m] --> AKG[m] + CO2 + NADH[m] |
| NADP-Isocitrate dehydrogenase (IDP1) | RHTO_04315/M7WN97 | ICI[m] + NADP[m] --> AKG[m] + CO2 + NADPH[m] |
| α-ketoglutarate dehydrogenase (KGD1) | RHTO_07893/M7WR40;  RHTO_04225/M7WPA9 | AKG[m] + NAD[m] --> SUCC-CoA[m] + NADH[m] + CO2 |
| Succinyl-CoA ligase (LSC1,2) | RHTO_01205/ 7WVW2;  RHTO_05264/M7WM30 | SUCC-CoA[m] + ADP <==> SUCC[m] + ATP |
| Succinate dehydrogenase complex (SDH1-4) | RHTO_00723 / M7XF32 | SUCC[m] + FAD[m] <==> FUM[m] + FADH[m] |
| Fumarate hydratase (FUM1) | RHTO_05746/M7XEU6 | FUM[m] <==> MAL[m] |
| Malate dehydrogenase (MDH1) | RHTO_04363/M7XHF8 | MAL[m] + NAD[m] <==> OAA[m] + NADH[m] |
| NADP-Malic enzyme (MAE1) | RHTO_07218/M7XHV2 | MAL[m] + NADP --> PYR[m] + NADPH[m] + CO2 |
| **Acetyl-CoA formation in cytosol** | | |
| Pyruvate decarboxylase (PDC1) | RHTO_00098/M7X6G2 | PYR[c] --> CO2 + ACETAL[c] |
| Mg(2+)-ACDH; Mg(2+)-activated acetaldehyde dehydrogenase (ALD6) | RHTO_05838 /M7WKL7 | NADP[c] + ACETAL[c] --> Acetate[c] + NADPH[c] |
| Acetyl-CoA synthetase (ACS2) | RHTO_08027/M7XFR0 | ATP + Acetate[c] --> AMP + AcCoA[c] |
| ATP:citrate lyase (ACL1) | RHTO_03915/M7WHC9 | CIT[c] + ATP --> AcCoA[c] + ADP + OAA[c] |
| **Anaplerotic and gluconeogenic reactions** | | |
| Pyruvate carboxylase (PYC1) | RHTO_02628/M7WS17 | PYR[c] + ATP + CO2 --> OAA[c] + ADP |
| Phosphoenolpyruvate carboxykinase (PCK1) | RHTO_07480/M7XSC4 | OAA[c] + ATP --> PEP[c] + ADP + CO2 |
| Fructose-1,6-bisphosphatase (FBP1) | RHTO_03046 /M7XYF6 | FBP[c] --> F6P[c] |
| **Glyoxylate cycle** | | |
| Isocitrate lyase (ICL1,2) | RHTO_05768/M7WLD5;  RHTO_03507/M7WQF1 | ICI[c] --> Glyoxy[c] + SUCC[c] |
| Malate synthase (MLS1,2) | RHTO_01459/M7WMR9 | Glyoxy[c] + AcCoA[c] --> MAL[c] |
| **Glycerol uptake** | | |
| Glycerol kinase (GUT1) | RHTO_07649/M7WRY8 | Glycerol[c] + ATP --> GLYC3P[c] + ADP |
| Mitochondrial Glycerol-3-phosphate dehydrogenase (GUT2) | RHTO_07665/M7XSX2 | GLYC3P[c] + FAD[m] --> DHAP[c] + FADH[m] |
| **D-xylose uptake** | | |
| Xylose reductase (XYL1) | RHTO_03963/M7X8C7 | Xylose[c] + NADPH[c] <==> Xylitol[c] + NADP[c] |
| Xylitol dehydrogenase (XDH) | RHTO_01970/M7WT79 | Xylitol[c] + NAD[c] <==> D-Xylulose[c] + NADH[c] |
| Xylulokinase (XKS1) | RHTO_04556/M7X6R2 | D-Xylulose[c] + ATP --> XYL-5P[c] + ADP |
| **L-arabinose uptake** | | |
| Arabinose reductase (AR) | RHTO_07387 /M7WCH0 | Arabinose[c] + NADPH[c] <==> arabitol[c] + NADP[c] |
| L-arabitol 4-dehydrogenase (LAD) | RHTO_01629/M7XCQ6 | arabitol[c] + NAD[c] <==> L-Xylulose[c] + NADH[c] |
| L-Xylulose reductase (LXR) | RHTO_00373/M7X791 | L-Xylulose[c] + NADPH[c] <==> Xylitol[c] + NADP |
| **TAG synthesis** | | |
| Fatty acid (C16) net reaction | -- | (8) AcCoA[c] + (7) ATP + (14) NADPH[c] --> C16-PAL[c] + (7) ADP + (14) NADP |
| TAG (C51) net reaction | -- | GLYC3P[c] + (3) C16-PAL[c] --> TAG[c] |
| **Oxidative phosphorylation and ATP maintenance** | | |
| NADH dehydrogenase and ATP synthase (NDE1, ATP1,2,16) | RHTO_00388/M7X7A4;  RHTO_03206/M7WXX4 | (20) NADH[m] + (24) ADP + (10) O2 --> (20) NAD[m] + (24) ATP |
| Succinate dehydrogenase complex (SDH1-4) | RHTO_00534/M7X6W8; RHTO_03206/M7WXX4 | (20) FADH[m] + (24) ADP + (10) O2 --> (20) FAD[m] + (24) ATP |
| ATP maintenance | -- | ATP --> ADP + ATPmaintenance[c] |
| Adenylate kinase (ADK1,2) | RHTO_02749/M7XXM8 | AMP + ATP --> (2) ADP |
| **Cytosolic reactions and membrane transport reactions** | | |
| Malate dehydrogenase | RHTO_03745/M7WQ86 | NADH[c] + OAA[c] <==> NAD[c] + MAL[c] |
| Fumarate hydratase | RHTO_05746/M7XEU6 | MAL[c] <==> FUM[c] |
| Fumarate reductase | RHTO_01560/M7WUD3 | NADH[c] + FUM[c] --> NAD[c] + SUCC[c] |
| Citrate synthase | RHTO_06406/M7XE29 | AcCoA[c] + OAA[c] --> CIT[c] |
| Isocitrate dehydrogenase | RHTO_01290/M7WW42 | ICI[c] + NADP --> AKG[c] + NADPH[c] + CO2 |
| Aconitate hydratase | RHTO_00539/M7X6X3 | CIT[c] <==> ICI[c] |
| Malic enzyme | RHTO_03795/M7WHN9 | MAL[c] + NADP --> PYR[c] + NADPH[c] + CO2 |
| Mitochondrial pyruvate carrier | RHTO_02579/ M7WRE9 | PYR[c] <==> PYR[m] |
| Mitochondrial OAA transporter | RHTO_00349/ M7X6E4 | OAA[c] <==> OAA[m] |
| Mitochondrial dicarboxylate transporter | RHTO_07389/M7WJ77 | MAL[c] <==> MAL[m] |
| Carnitine-O-acetyltransferase (active only on non-sugar substrates) | RHTO_01903/M7WLQ0 | AcCoA[c] --> AcCoA[m] |
| Mitochondrial citrate transporter | RHTO_03384/M7WRF8 | CIT[c] + MAL[m] <==> CIT[m] + MAL[c] |
| Mitochondrial citrate transporter | RHTO_03384/M7WRF8 | ICI[m] + CIT[c] <==> ICI[c] + CIT[m] |
| Mitochondrial succinate-fumarate transporter | RHTO_05007/M7WW62 | SUCC[c] + FUM[m] --> FUM[c] + SUCC[m] |
| Mitochondrial dicarboxylate transporter | RHTO_07389/M7WJ77 | SUCC[c] --> SUCC[m] |
| Mitochondrial AKG carrier | -- | AKG[m] <==> AKG[c] |
| NAD-NADH shuttle | -- | NADH[c] + NAD[m] --> NADH[m] + NAD[c] |
| **Sulphate assimilation** | | |
| Sulphate uptake | RHTO_00048 /M7X6A7 | SO4[c] + (4) NADPH[c] + (3) ATP --> H2S[c] + (4) NADP + (3) ADP |
| **Ammonia assimilation** | | |
| Glutamate synthetase (GLT1) | RHTO_00025/M7WY92 | AKG[c] + GLUM[c] + NADH[c] --> (2) GLUT[c] + NAD[c] |
| Glutamine synthetase (GLN1) | RHTO_00673/M7XEY4 | ATP + NH3[c] + GLUT[c] --> GLUM[c] + ADP |
| **Biomass formation** | | |
| Biomass formation | -- | (10) OAA[c] + (6) 3-PG[c] + (3.2) Ribose-5P[c] + (254) ATP + (90) NADPH[c] + (22) NADPH[m] + (16) NAD[c] + (6) NAD[m] + (24) AcCoA[c] + (3) AcCoA[m] + (18) PYR[c] + (11) AKG[m] + (3.2) E4P[c] + (6.2) PEP[c] + (1.53) H2S[c] + (4.5) F6P[c] + (25) G6P[c] + GLYC3P[c] --> biomass[c] + (22) NADP + (90) NADP + (16) NADH[c] + (6) NADH[m] + (254) ADP |
| Hypothetical NADP-transhydrogenase (sthA) | RHTO_06438 | NADP[c] + NADH[c] <==> NADPH[c] + NAD[c] |

**Comparison of solution space on different substrates obtained by Elementary mode analysis**

0.63

0.56

0.50

0.58

0.53

0.32

0.55

0.45

**Figure S1:** The biomass yield [c-mol.c-mol^-1^] on corresponding substrates is plotted against the TAG yield on the substrate which is in c-mol.c-mol^-1^. Theoretical maximum yields on each substrate with and without biomass formation are represented.


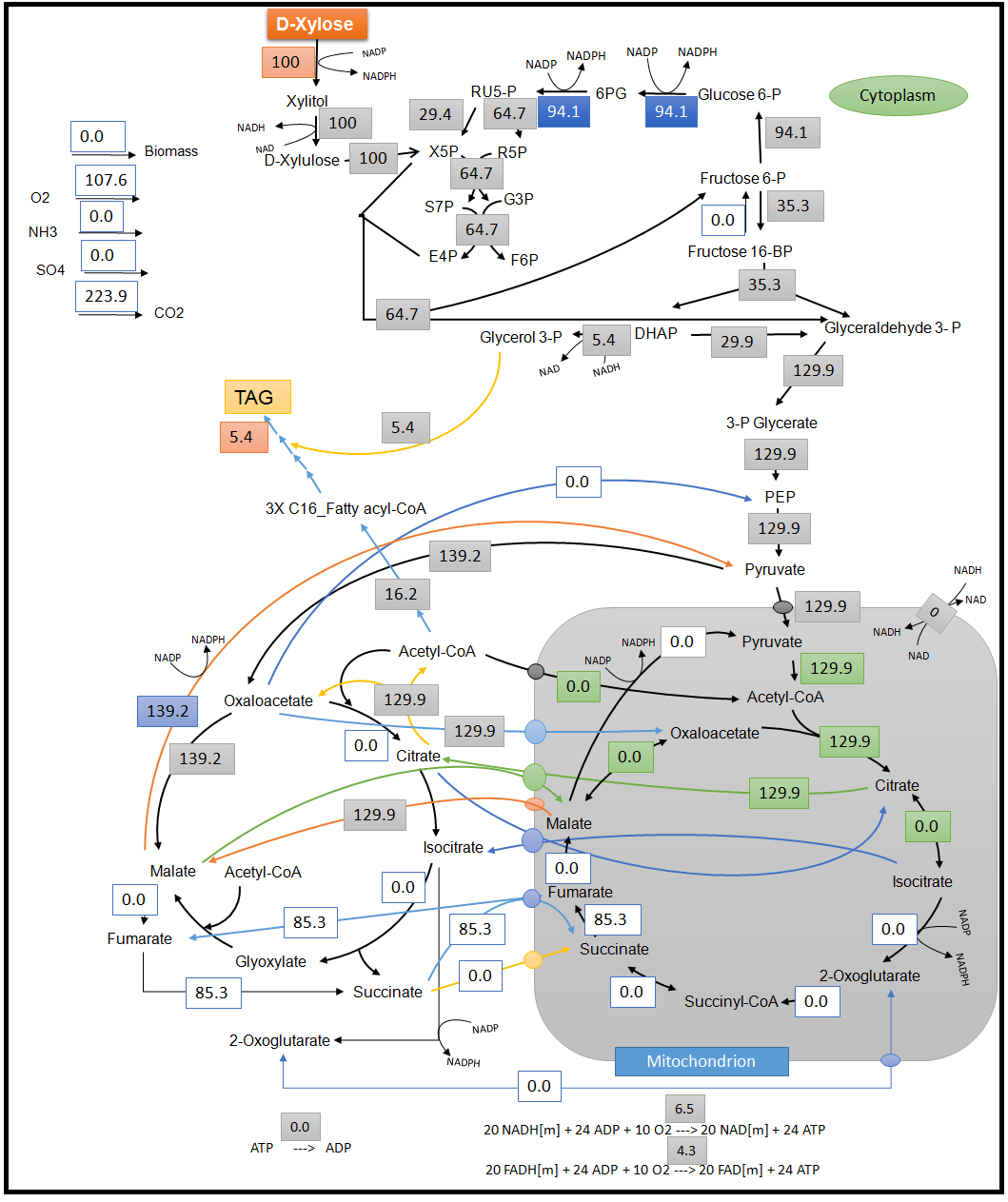


Figure S2: Optimal flux distribution on xylose. All values are relative molar fluxes (mmol.g^-1^h^-1^) normalized to the xylose uptake rate


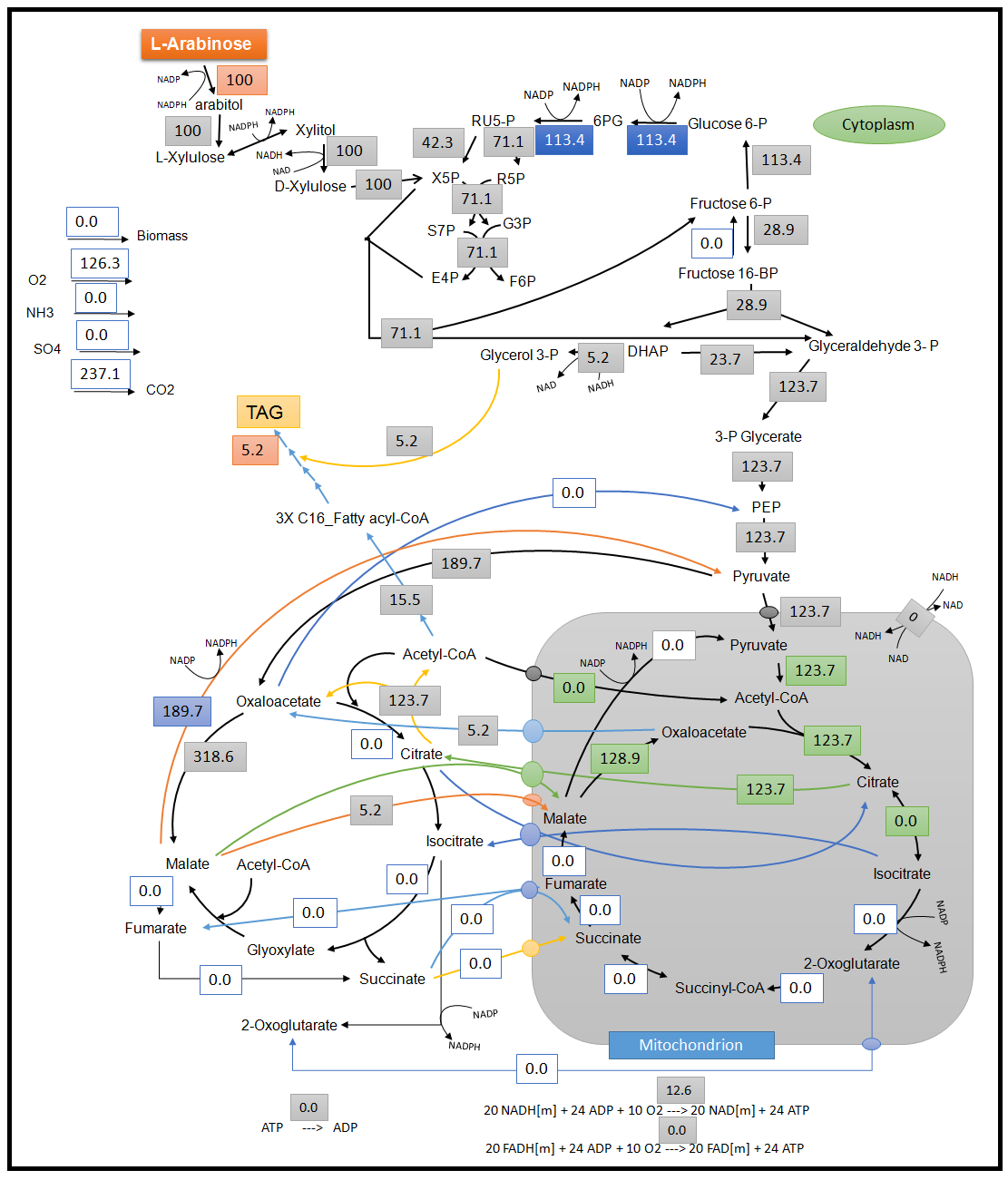


Figure S3: Optimal flux distribution on arabinose. All values are relative molar fluxes (mmol.g^-1^h^-1^) normalized to the arabinose uptake rate


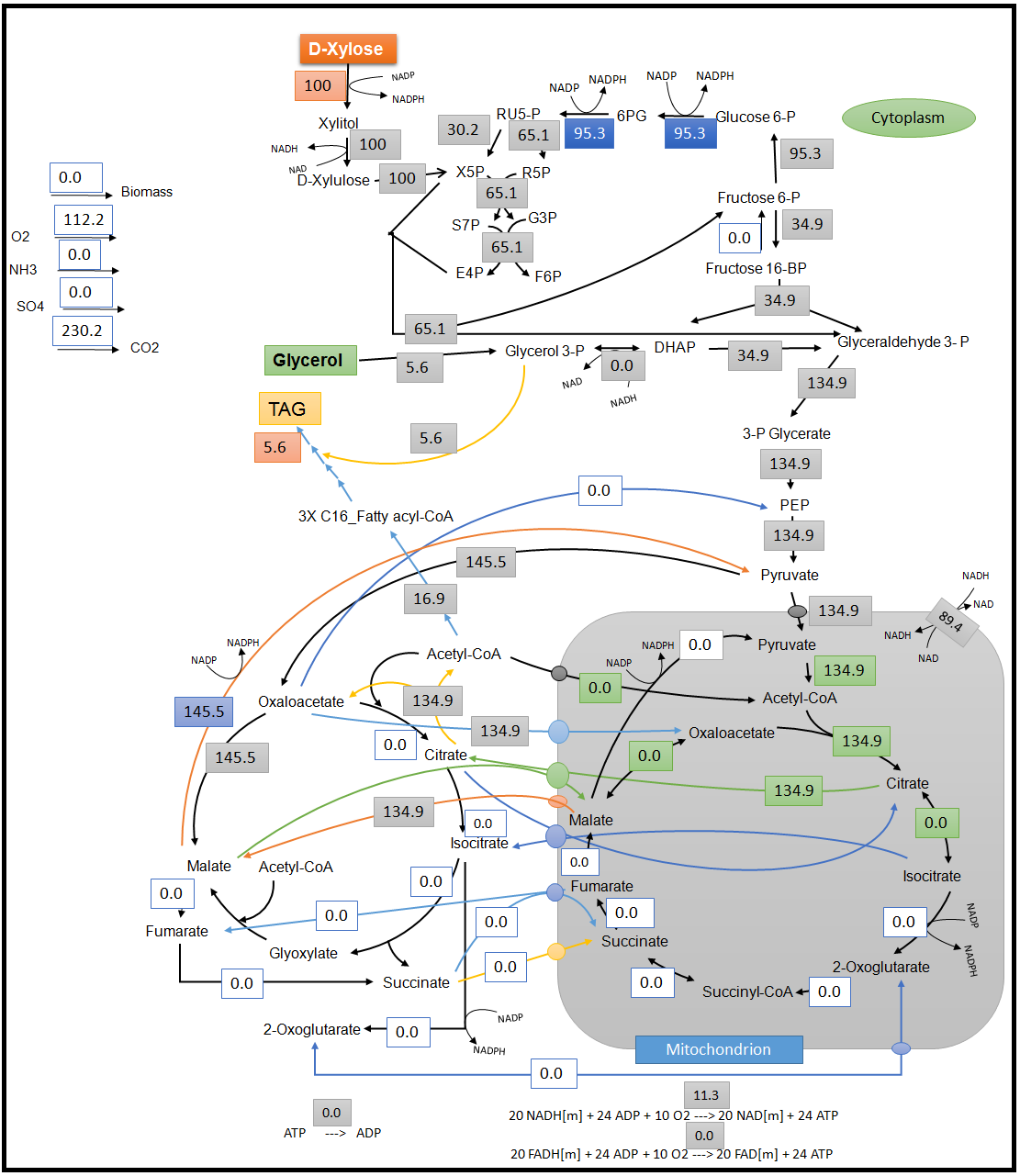


Figure S4: Optimal flux distribution on xylose and glycerol. All values are relative molar fluxes (mmol.g^-1^h^-1^) normalized to the xylose uptake rate


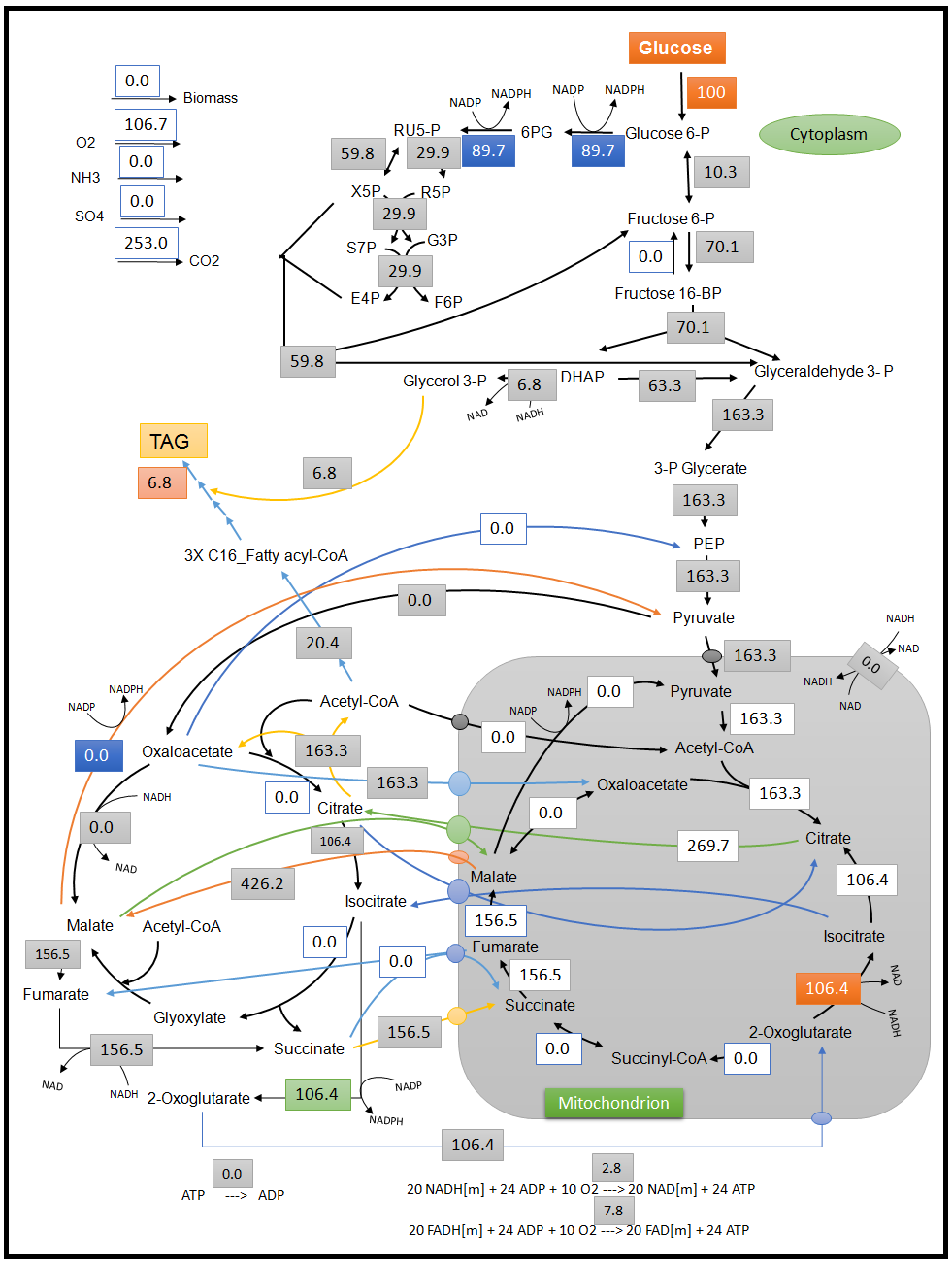


Figure S5: Optimal flux distribution on glucose with ICDH acting in reverse direction and without cytosolic malic enzyme. All values are relative molar fluxes (mmol.g^-1^h^-1^) normalized to the xylose uptake rate

**References**

1. Liu H, Zhao X, Wang F, Li Y, Jiang X, Ye M, Zhao ZK, Zou H. **Comparative proteomic analysis of Rhodosporidium toruloides during lipid accumulation**. *Yeast* 2009**,26**: 553-566.
2. Zhu Z, Zhang S, Liu H, Shen H, Lin X, Yang F, Zhou YJ, Jin G, Ye M, Zou H, Zhao ZK. **A multi-omic map of the lipid-producing yeast Rhodosporidium toruloides**.2012, *Nat Commun* **3**: 1112.
3. Kumar S, Kushwaha H, Bachhawat AK, Raghava GP, Ganesan K**.** **Genome sequence of the oleaginous red yeast *Rhodosporidium toruloides* MTCC 457.**2012, *Eukaryot Cell:* **11:**1083-1084.
4. Gruchattka E, Hadicke O, Klamt S, Schutz V, Kayser O. **In silico profiling of Escherichia coli and Saccharomyces cerevisiae as terpenoid factories**.2013, *Microb Cell Fact* **12**: 84.
